# The Matrix

**Published:** 1905-09

**Authors:** F. L. Fossume

**Affiliations:** New York


### THE MATRIX.[1]

BY F. L. FOSSUME, NEW YORK.

Mr. Chairman and Members of the Institute of Stomatology,—At the request of your Executive Committee it is my pleasure to-night to describe to you my way of constructing the inlay matrix. I will do this as briefly as possible and not elaborate upon the various methods which from time to time have been recommended. Simplicity and practical utility of method must always be the aim, and although it usually takes but a few minutes to construct a matrix, there are occasionally cavities from which I find it very difficult to obtain a perfect one. At the clinic before the International Dental Congress I made eleven matrices, and, without haste, consumed between five and seven minutes for each. Dr. Stanton was the patient and the inlay can be seen in his tooth.

Rolled gold and platinum are the metals used. Gold is superior to platinum by being more pliable and not adhering to the backed porcelain; it can only be used with a body fusing below 1900°. I prefer No. 60, except in very small cavities, where 30 may be used. Platinum has no peer on account of its power to resist heat and for holding its shape against the pulling of the shrinking porcelain body when fusing; one-one-thousandth, one-twelve-hundredth, and one-two-thousandth of an inch in thickness are used.

When the cavity is prepared, a piece of metal of proper size is cut, and this is of some importance. For large contours it is well to have as much of the form of the tooth outlined by the matrix as possible, so as to guide one when building up the inlay. In other cavities, especially between teeth, broad laps will prevent the withdrawal of the matrix and otherwise be in the way.

---

[1] Read before The New York Institute of Stomatology, May, 1905.

For adjusting the thin metal in place in the cavity I have found spunk the best. A piece more than large enough to fill the whole cavity is placed over the metal and pressed gently against it, driving it in until it is well against the floor, and then considerable pressure is used on the spunk so as to swage the metal in place as well as possible. The spunk is now removed and a band of rubber dam is stretched over the matrix and tooth and the ends held by an assistant or the left hand. The matrix can be seen through the stretched transparent dam, held firmly against the edges everywhere, and it will not rock under the final burnishing. For this purpose I use ivory points; my set is composed of ten instruments, which I will pass around for your inspection. You will notice that some have flat points and sharp edges; this is to fit the form of the cavity, which I always strive to have with reasonably flat floor and straight walls or sides. I wish to call your attention to No. 6, which is for finishing the edges, and No. 8 for pressing the inlay in position when setting with cement and in very large cavities for supporting the matrix with the spunk. Gum camphor is often of great help for swaging the matrix into the cavity, as it can be burnt out of the matrix when removed, or dissolved with alcohol while in the cavity, if it is pressed in so tight as to prevent the removal of the matrix.

When the restoration is to be exceptionally large, and when it is very difficult to support the matrix so as to prevent rocking, I burnish the floor and one wall and then bake some porcelain into this, then reinsert in cavity, and, while the other portions are being burnished, this porcelain serves as a support.

The whole secret in porcelain inlay work is the preparation of the cavity, as this must be prepared in such a manner that the perfect matrix can be obtained from it, and at the same time it must support the inlay with the same strength and accuracy as in mosaic work.

If you with reasonable care have tried to construct a matrix and failed to get a satisfactory result, scrutinize your cavity carefully and you will see that the fault lies there and not with your matrix. If your cavities are correctly prepared, it is practically impossible not to obtain a satisfactory matrix, assuming reasonable skill.

